# Association at Savannah

**Published:** 1875-05

**Authors:** 


### Editorial.

---

We occupy considerable space in the present number of the Journal with the reports sent us from the Medical Associations of Alabama and Georgia at their recent annual meetings. Whilst these reports are necessarily brief, and less complete than we could desire, they will nevertheless be read with interest by those of our readers whose professional duties have deprived them of the privilege of attendance upon the meetings.

Although in attendance in person at the Georgia Association, our several official duties deprived us of the opportunity of hearing and enjoying the major part of the essays read and the discussions thereon. We are warranted, however, in saying that the attendance at Savannah, though not the largest in the history of the Association, reached nearly or quite one hundred members, and embraced an unusual number of what might be termed really representative men of the profession of the State.

The hospitality of the Savannah profession was most cordial, and manifested in a free-handed profusion, the animus of which we are obliged both to admire and appreciate; and yet we must be permitted to say that we *deprecate* these costly entertainments of our State Associations. In the acceptance of such hospitalities we feel always a personal constraint which adds nothing to the enjoyment of the occasion, but rather detracts from it. That there are any who follow in the wake of our Association with an eye to the loaves and fishes, or even to the sparkling nectar which exhilarates the brain and loosens the tongue, we are loath to believe. The Georgia profession, in the aggregate, is impecunious to a degree, and we know of none who can be esteemed in any sense wealthy. The money lavished upon these annual assemblages, though freely and heartily given, cannot be viewed otherwise than a burden upon those who give, and a like burden upon those who accept it. In view of the fact that a great effort has been, and is being, made all over the State to stay the tide of sad havoc which intemperance is yearly making amongst the best of our population—and what class is suffering more than our own profession—it is pertinent to ask, is it not the duty of our medical bodies, in their festivities, to withhold the intoxicating cup and stand neutral, at least, upon the great question involved? We are glad to see that the Committee of Arrangements for the approaching meeting of the American Medical Association at Louisville have so thought and determined.

Georgia's venerable metropolitan city by the seaside, bedecked in the effulgence of blossoming spring time, redolent with the perfume of its semi-tropical garlands, embowered beneath the nurturing boughs of her antiquated oaks, sporting in her youthful hilarity through the parks and shaded squares which invite one's tarrying at every turn, was never more beautiful and enchanting than now. The air of quiet luxury and ease which pervades the corridors and drawing-rooms of so many of her princely mansions, everywhere indented by the finger of time, tempts one to a most welcome unbending from the tension of our daily treadmill toil, to a wide throwing open of the secret chambers of the heart in grateful response to the lavished hospitalities of her generous-hearted citizens. The occasion could not be other than one of delightful recreation and real, hearty enjoyment.

The number of essays read at the meeting was large. Many of them evidenced mature thought, and were listened to by an attentive auditory. It was pleasant to see the harmony of the occasion unbroken by a ripple upon the surface, and the whole time of the body given to the scientific and professional objects of its assemblage. May it always be so.

The next meeting was set down for Augusta with a pleasant unanimity, and a tacitly implied pledge given for Macon in the following year.

